# Developmental Distribution of the Plasma Membrane-Enriched Proteome in the Maize Primary Root Growth Zone

**DOI:** 10.3389/fpls.2013.00033

**Published:** 2013-03-06

**Authors:** Zhe Zhang, Priyamvada Voothuluru, Mineo Yamaguchi, Robert E. Sharp, Scott C. Peck

**Affiliations:** ^1^Division of Biochemistry, University of MissouriColumbia, MO, USA; ^2^Christopher S. Bond Life Sciences Center, University of MissouriColumbia, MO, USA; ^3^Interdisciplinary Plant Group, University of MissouriColumbia, MO, USA; ^4^Division of Plant Sciences, University of MissouriColumbia, MO, USA

**Keywords:** plasma membrane, proteomics, growth zone, maize, roots, development

## Abstract

Within the growth zone of the maize primary root, there are well-defined patterns of spatial and temporal organization of cell division and elongation. However, the processes underlying this organization remain poorly understood. To gain additional insights into the differences amongst the defined regions, we performed a proteomic analysis focusing on fractions enriched for plasma membrane (PM) proteins. The PM is the interface between the plant cell and the apoplast and/or extracellular space. As such, it is a key structure involved in the exchange of nutrients and other molecules as well as in the integration of signals that regulate growth and development. Despite the important functions of PM-localized proteins in mediating these processes, a full understanding of dynamic changes in PM proteomes is often impeded by low relative concentrations relative to total proteins. Using a relatively simple strategy of treating microsomal fractions with Brij-58 detergent to enrich for PM proteins, we compared the developmental distribution of proteins within the root growth zone which revealed a number of previously known as well as novel proteins with interesting patterns of abundance. For instance, the quantitative proteomic analysis detected a gradient of PM aquaporin proteins similar to that previously reported using immunoblot analyses, confirming the veracity of this strategy. Cellulose synthases increased in abundance with increasing distance from the root apex, consistent with expected locations of cell wall deposition. The similar distribution pattern for Brittle-stalk-2-like protein implicates that this protein may also have cell wall related functions. These results show that the simplified PM enrichment method previously demonstrated in *Arabidopsis* can be successfully applied to completely unrelated plant tissues and provide insights into differences in the PM proteome throughout growth and development zones of the maize primary root.

## Introduction

Unlike animals, plants continue to grow and increase in size throughout their lifecycle. Plant growth, however, does not occur indiscriminately but rather is distributed through defined regions of roots and shoots that are referred to as the growth zones (Erickson and Silk, 1979). Within these regions of growth, there is considerable variability in spatial and temporal cell production and cell elongation, two processes defining the overall growth rate (Peters and Bernstein, 1997; Beemster and Baskin, 1998). Therefore, these growth zones have been used extensively to study the processes contributing to cell production and elongation (Pilet et al., 1983; MacAdam et al., 1992; Bernstein et al., 1993; Liang et al., 1997).

Among plant organs, the root has a relatively simple organization of the growth zone, making it a good model for studying various aspects of plant growth (Beemster and Baskin, 1998; Sharp et al., 2004; Brady et al., 2005). The meristematic region with dividing cells is near the tip of the root, and the elongation region consisting of cells at various stages of expansion progresses away from the zone of division. Cell elongation accelerates as cells move out of the meristem, reaching a maximal rate in the middle of the growth zone, and decelerates thereafter such that cells reach their final length by the end of the growth zone. This spatial distribution of cell production and cell elongation has been exploited for numerous studies of growth related processes, revealing gradients within these zones of metabolites, phytohormones, pH, transcription factor proteins, and cell wall-modifying proteins (Mulkey and Evans, 1980; Pilet et al., 1983; Sharp et al., 1990; Baskin et al., 2004; Brady et al., 2005; Wolters and Jurgens, 2009; Yamaguchi and Sharp, 2010).

Because the plasma membrane (PM) is the interface between a cell and the apoplast/environment, it is a key structure involved in integrating signals and responses involved in cell growth. For instance, PM-localized proteins such as ion and water channels are necessary for regulating turgor and cell elongation in roots (Kiegle et al., 2000; Hachez et al., 2006). The acidification of apoplastic pH by the PM-localized proton ATPase has been shown to be important for wall loosening and cell elongation in different plant species (Pilet et al., 1983; Cosgrove, 1989). Additionally, cells at various stages of elongation must constantly modify their cell walls, and several metabolites needed for cell wall deposition are transported across the PM (Wightman and Turner, 2010; Endler and Persson, 2011).

Despite the important functions of PM proteins in the regulation of growth, these proteins are often underrepresented in proteomic analyses because they are found in low concentrations relative to the total cellular protein content (Ephritikhine et al., 2004; Morel et al., 2006; Zhang and Peck, 2011). To increase depth of coverage, aqueous two-phase partitioning is often utilized which, although ideal for yielding highly purified PM fractions, is both time-consuming and technically challenging, often requiring significant technical optimization for each new tissue or species examined (Albertsson et al., 1987; Komatsu, 2008). Although crude microsomal fractionation does enrich for PM proteins over total protein, the large degree of contamination from other organellar proteins, particularly from the endoplasmic reticulum (ER), still limits the depth of coverage of PM proteomes. Using *Arabidopsis* suspension cell cultures as starting material, Zhang and Peck (2011) recently reported a simple method for decreasing the representation of organellar proteins from crude microsomal fractions to obtain greater than threefold enrichment of PM proteins from *Arabidopsis* culture cells for proteomic analyses while decreasing contamination with ER proteins by sevenfold. Although this method is not applicable to assigning definitive location of a protein to the PM because it does not yield samples as pure as those from aqueous two-phase partitioning, the strategy is useful to enrich sufficiently for the PM fraction to allow for meaningful quantitative comparisons. In the current study, we evaluated the applicability of this simplified PM enrichment method using the growth zone of maize primary roots grown under well-watered conditions. We demonstrate that the strategy is easily transferred to this new tissue and species, and we report the region-specific distribution of proteins, including many PM proteins, associated with the spatial growth pattern.

## Materials and Methods

### Chemicals

All chemicals used in this study were ultrapure grade (obtained from Sigma-Aldrich Co, St. Louis, MO, USA; Fisher Scientific, Pittsburgh, PA, USA; Promega Corp., Madison, WI, USA). HPLC-water was obtained from the Millipore Synthesis system (Millipore Corp. Billerica, MA, USA).

### Plant growth and tissue collection

**Figure 1:**
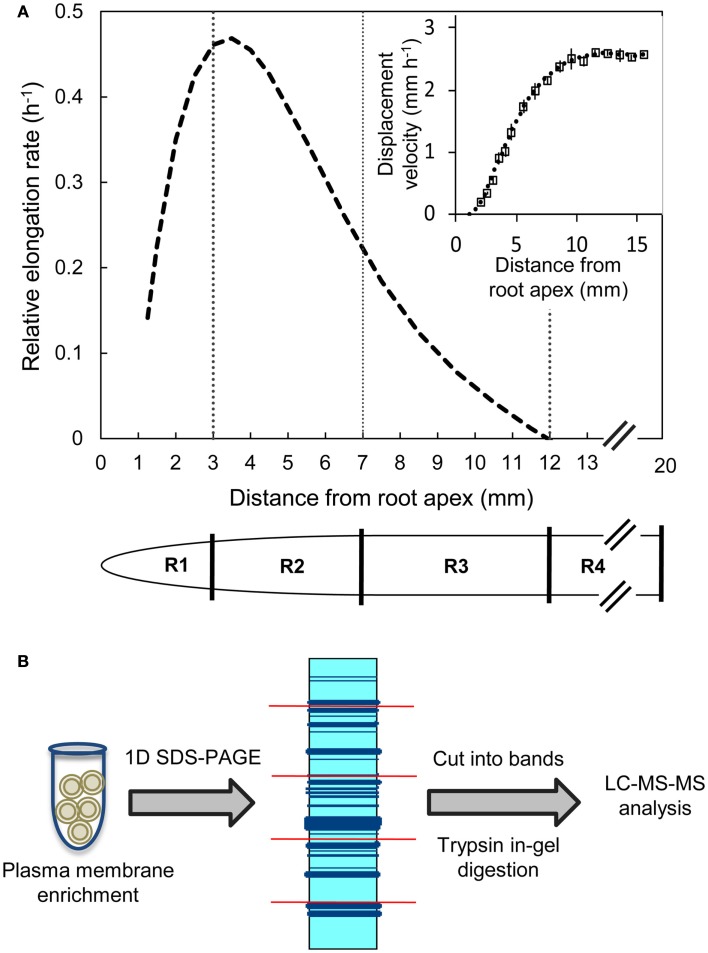
**(A)** Relative elongation rate profile and displacement velocity profile (inset) as a function of distance from the root apex (including the root cap) of the primary root of maize hybrid line B73 X MO17 grown under well-watered conditions. The spatial distribution of displacement velocity (mm h^−1^) from the root apex was calculated from root elongation rate and cell length profile data obtained at 48 h after transplanting (data are means ± SE, *n* = 3). A fifth-order polynomial curve was fitted to the displacement velocity profile and the derivative of displacement velocity against position was used to obtain the relative elongation rate profile (h^−1^). Tissues from region 1 (R1, 0–3 mm), region 2 (R2, 3–7 mm), region 3 (R3, 7–12 mm), and region 4 (R4, 12–20 mm) were harvested. **(B)** Label-free proteomics work flow shown for plasma membrane-enriched samples as protein input followed by in-gel tryptic digestion and LC-MS/MS analysis.

B73 X MO17 hybrid maize seed were used in all experiments. Seeds were surface sterilized in 5% NaClO solution for 15 min, rinsed with deionized water for 15 min, and imbibed in aerated 1 mM CaSO_4_ solution for 24 h. The imbibed seeds were germinated between sheets of germination paper moistened with 1 mM CaSO_4_ solution at 29°C and near-saturating humidity in the dark. Seedlings with primary roots of 10–20 mm in length were transplanted against the interior surface of Plexiglass containers filled with vermiculite (no. 2A, Therm-O-Rock East Inc., New Eagle, PA, USA) which was moistened to the drip point with 1 mM CaSO_4_ solution. The seedlings were then grown at 29°C and near-saturating humidity in the dark for 48 h. Primary root elongation was monitored by periodically marking the position of the root apices on the Plexiglass. The apical 20 mm of the primary roots were harvested and divided into four regions (all distances are from the root apex including the root cap); the 0–3 mm region (R1), the 3–7 mm region (R2), the 7–12 mm region (R3), and the 12–20 mm region (R4) (Figure 1A). The harvested root segments were collected by position, transferred to tubes containing liquid nitrogen, and stored at −80°C. In each of three replicate experiments, root segments were harvested from >150 seedlings for proteomic analysis. Transplanting, root elongation measurements, and harvesting were performed using a green “safe” light (Saab et al., 1990).

### Kinematic analysis of displacement velocity and relative elongation rate profiles

The spatial distributions of displacement velocity and relative elongation rate in the primary root growth zone were obtained from cell length profiles and root elongation rates as described by Silk et al. (1989). Briefly, 10 seedlings were grown for 48 h and their root elongation monitored periodically. Elongation rates were steady after ∼15 h from transplanting as required for accurate determinations of elongation rate profiles from anatomical records. Three to four roots that were straight and had an elongation rate similar to the mean of the population were then harvested for cell length measurements. The apical 15 mm was sectioned longitudinally using a vibratome (Lancer series 1000 Vibratome, St Louis, MO, USA) and a 125 μm thick section of each root was stained with 1 mg mL^−1^ of Calcofluor (Sigma-Aldrich, St. Louis, MO, USA) for 15 min to visualize the cell walls. The stained sections were imaged by confocal microscopy as described by Yamaguchi et al. (2010). Average cell lengths at various positions from the root apex were calculated by measuring the cell lengths of 4–12 cortical cells. The final cell lengths were calculated by averaging the four most distal measurement positions (12–15 mm from the root apex). Displacement velocities were calculated using the relationship *L*_A_/*L*_F_ = *V*_A_/*V*_F_, as described in Silk et al. (1989) where

*L*_A_ = the mean cell length at position A
*L*_F_ = final cell length
*V*_A_ = displacement velocity at position A
*V*_F_ = the final displacement velocity (equal to the root elongation rate).

The cell length method cannot be used to calculate accurate displacement velocities in the meristematic region (Silk et al., 1989). Therefore, displacement velocities were calculated starting at the distal end of the meristem, which was estimated to occur at a cell length of 2.5 times the length of the shortest cells (2.5 mm from the root apex; Erickson, 1961). The mean displacement velocities were plotted and a fifth-order polynomial curve was fitted to the data (Figure 1A inset). The derivative of the resulting curve was used to obtain the relative elongation rate profile (Figure 1A; Silk et al., 1989).

### Protein extraction and plasma membrane enrichment

Frozen root tissues were ground to a fine powder and proteins were extracted as described by Zhang and Peck (2011). Briefly, proteins were extracted from the tissues using 1 mL of ice-cold buffer H (330 mM sucrose, 50 mM HEPES/KOH pH 7.5, 50 mm Na_4_P_2_O_7_, 25 mm NaF, 5% glycerol, 0.5% polyvinyl pyrrolidone, 10 mm EDTA, 1 mM Na_2_MoO_4_, 1 mM PMSF, 10 mM leupeptin A, 1 nM calyculin A, and 3 mM DTT) per gram fresh weight of tissue. The samples were subsequently centrifuged for 10 min (10,000 × *g* at 4°C) to remove cell debris. The supernatant was used to obtain crude microsomal pellets by ultracentrifugation for 30 min (100,000 × *g* at 4°C). The microsomal pellets were washed with buffer H without DTT, incubated in 2 μL of buffer B (buffer H without DTT but with 0.02% w/v Brij-58) per μg of crude microsomal protein on ice for 45 min and centrifuged for 30 min at 100,000 × *g*. The resulting pellets were washed with 10 mM Na_2_CO_3_ (pH 11–12) in buffer H without DTT to yield the PM-enriched protein fraction.

### Immunoblotting

Protein samples from the soluble fraction, crude microsomes, and PM-enriched fraction were separated by SDS-PAGE and transferred to Immobilon P membrane for 2 h at 70 V in Towbin’s transfer buffer (39 mM glycine, 48 mM Tris-base, 0.037% SDS, and 20% methanol) at 4°C. The membranes were incubated first in blocking buffer (Tris-buffered saline at pH 7.8 containing 5% non-fat milk and 0.05% Tween-20) for 1 h at room temperature, then incubated with primary antibodies in blocking buffer at 4°C overnight, and finally incubated in blocking buffer containing the secondary antibody horseradish peroxidase-labeled anti-rabbit IgG (Sigma-Aldrich Co, St. Louis, MO, USA) for 1 h at room temperature. Supersignal Femto or Pico substrate (Thermo Scientific, IL, USA) was used for chemiluminescence detection. The primary antibodies (antibodies were obtained from Agrisera, Sweden) used in the study were α-AHA (H^+^-ATPase, PM marker) and α-SMT-1 (sterol methyltransferase-1, ER marker).

### SDS-PAGE analysis and tryptic digestion

For each biological replicate, equal concentrations (∼30 μg) of PM-enriched protein from each region of the growth zone were solubilized in sample loading buffer, heated to 75°C, separated by 8% SDS-PAGE and stained with colloidal Coomassie G-250 overnight, as described by Neuhoff et al. (1985). The following day, gels were destained in distilled water, and each gel lane was cut into eight slices with a razor blade. The proteins from the gel slices were reduced with 50 mM TCEP-HCl, alkylated with 50 mM iodoacetamide and digested within the gel overnight using 1:20 w/w trypsin in 100 mM ammonium bicarbonate buffer (pH 8.3) at 37°C. After digestion, the peptides were eluted twice with 1% trifluoroacetic acid with 60% acetonitrile, and the eluted mixture was lyophilized overnight to obtain dried peptides. The dry peptides were stored at −80°C until LC-MS/MS analysis.

### LC-MS/MS analysis

Lyophilized peptides dissolved in 0.1% formic acid were applied to a 10 cm prepacked column (Picotips with 75 μm inner diameter and 15 μm tip, obtained from New Objective, Woburn, MA, USA) and eluted into the nanoelectrospray ion source of a LTQ-Orbitrap LC-MS/MS mass spectrometer (Thermoelectron Corp., Rockford, IL, USA) that was controlled by XCalibur version 2.2.1. The mass spectrometer operating in data-dependent mode was used to carry out a fully automated chromatography run using 1% formic acid and 99.9% acetonitrile, 0.1% formic acid with a 1% per min incremental gradient for the first 45 min and 11% per min for the final 5 min. The mass spectrometer measurements were obtained with the specifications as described in Zhang and Peck (2011).

### Peptide and protein identification

Mascot Distiller version 2.0 (Matrix Science, London, UK) was used to deconvolute the tandem mass spectra. However, de-isotoping was not performed. Mascot (server version 2.3, Matrix Science, London, UK) and X! Tandem (version 2007.01.01.1)^1^ were used to analyze the MS/MS spectra by searching an in-house database created by downloading all *Zea mays* proteins from NCBI^2^ and filtering for duplicate entries. The MS/MS based peptide and protein identities were validated by Scaffold (version Scaffold_3_00_08, Proteome Software Inc., Portland, OR, USA). The specified variable modifications were oxidized methionine and iodoacetamide derivative of cysteine. The identities of peptides (at greater than 95% probability) were accepted using the Peptide Prophet algorithm (Keller et al., 2002), and identities of proteins with at least two peptides identified (at greater than 99% probability) were accepted using the Protein Prophet algorithm (Nesvizhskii et al., 2003), respectively. If proteins containing similar peptides could not be differentiated based on MS/MS analysis alone, they were grouped to satisfy the principles of parsimony.

### Data analysis and bioinformatics

Spectral counts were normalized within a biological replicate using the mean of total spectral counts from all four regions (the average deviation within an experiment was ∼3%). For each region, the mean and standard deviation were calculated using the spectral counts from all three biological experiments. Proteins for which the fold difference between the means was greater than the two times the coefficient of variation (CV) determined for a technical replicate determined using these samples (CV = 0.34) was considered for pattern analysis.

**Figure 2:**
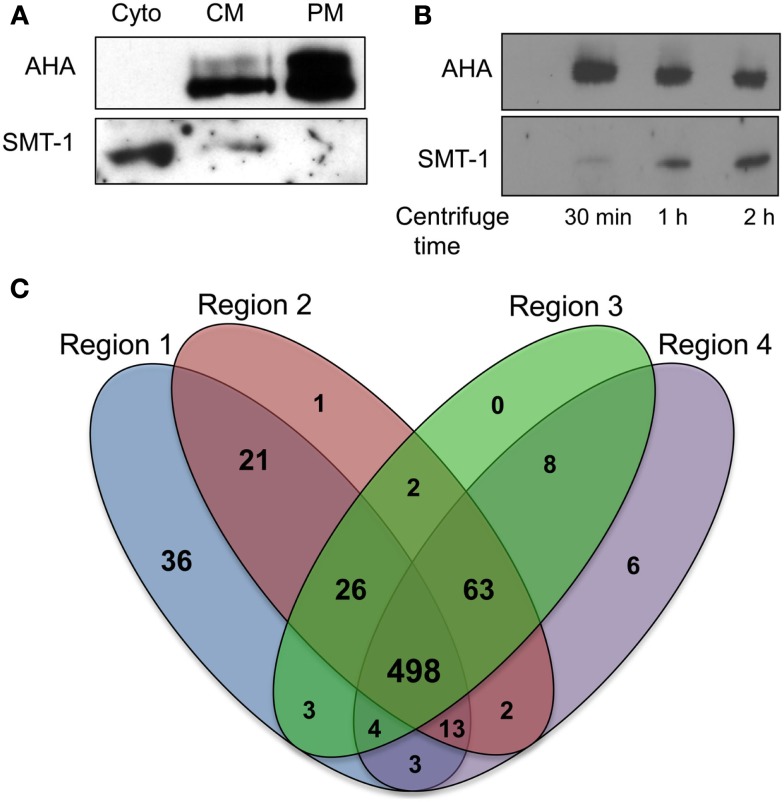
**Application of plasma membrane enrichment strategy to analysis of maize primary roots**. **(A)** Proteins from steps of the enrichment procedure were analyzed by immunoblotting with organelle specific markers (Cyto, cytosolic fraction; CM, crude microsomal fraction; PM, plasma membrane-enriched fraction). Equal amounts of proteins from each fraction were analyzed with antibodies specific for a plasma membrane H^+^-ATPase (AHA) or an endoplasmic reticulum integral membrane sterol methyltransferase (SMT-1). **(B)** Increasing time of ultracentrifugation increases ER proteins in crude microsomal pellets. Proteins were prepared for immunoblot analysis as for **(A)**, except that the ultracentrifugation at 100,000 × g was performed for 30 min, 1 h, or 2 h. Equal amounts of proteins from each crude microsomal fraction were analyzed with antibodies specific for PM H^+^-ATPases (AHA) or an endoplasmic reticulum integral membrane sterol methyltransferase (SMT-1). **(C)** Four-way Venn diagram showing number of proteins identified from the four regions (created using algorithm from: http://bioinfogp.cnb.csic.es/tools/venny/index.html).

To assign the distribution of proteins within the different regions, the protein data were analyzed according to the following steps. Proteins identified in at least two out of the three biological replicates within a region were considered reproducible. Reproducible proteins were used to generate a four-way Venn diagram (Figure 2C) using the algorithm at http://bioinfogp.cnb.csic.es/tools/venny/index.html. Proteins with a region-specific distribution pattern (i.e., with zero spectral counts in one or more region) were grouped into the three classes “single region present,” “single region absent,” and “two-region present” (Table 2). For proteins that were present in all regions, five major patterns of distribution were identified that were based on the gradient starting from R1 to R4, such as “decreasing,” “increasing,” “R1-lowest,” “R2-highest,” and “R3-highest” (Table 1). Proteins that were not present in all regions were grouped into three classes: “single region present,” “single region absent,” and “two regions present/absent” (Table 2).

**Table 1:** **Proteins present in all regions with distinct distribution patterns in the primary root growth zone**.

Protein name	Accession number	Mol. weight (kDa)	Mean NSC				Standard error				Pattern category	R1	R2	R3	R4
			R1	R2	R3	R4	R1	R2	R3	R4					
Cellulose synthase-2	gi|162459760	121	13.32	45.07	40.11	35.34	3.76	10.55	1.86	10.49	R1-lowest	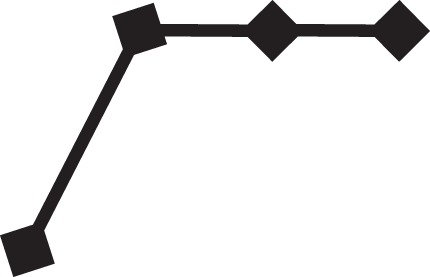			
Cellulose synthase-4	gi|162461169	121	5.97	34.07	25.09	26.85	1.15	11.10	3.33	7.48					
Cellulose synthase-8	gi|162460924	123	4.35	46.87	41.68	36.66	1.51	11.48	4.69	11.88					
Fasciclin-like Arabinogalactan protein 7 precursor	gi|195612412	27	3.63	8.83	8.32	8.32	0.62	0.61	1.10	1.43					
Brittle stalk-2-like protein 3	gi|119034638	50	3.96	9.65	11.63	10.17	0.94	1.48	1.86	1.57					
Glycoside transferase, six-hairpin, subgroup	gi|195614916	69	3.33	11.82	14.36	13.59	0.70	3.81	1.80	4.32					
Transmembrane 9 superfamily protein member 2 precursor	gi|195612428	74	16.67	19.34	16.67	14.34	2.73	1.33	4.84	1.86	R2-highest	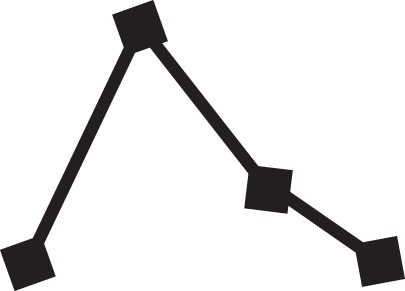			
Unknown	gi|194689992	72	11.19	15.45	11.09	8.98	2.82	2.95	3.64	1.38					
Unknown	gi|223948977	89	35.71	44.98	26.07	12.45	9.20	14.07	5.96	2.14					
Unknown	gi|223975481	72	3.67	5	4.34	1.05	0.67	0.58	0.88	0.12					
Unknown	gi|219884637	48	20.48	35.8	23.44	19.14	6.02	7.67	5.58	5.68					
Ubiquitin fusion protein	gi|902527	18	12.67	20.67	28.67	26	4.06	7.54	7.32	9.30	R3-highest	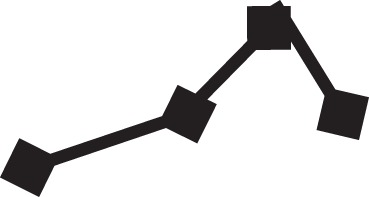			
Unknown	gi|194702312	54	43.36	51.06	59.13	54.49	4.64	3.66	2.25	8.67					
Unnamed protein product	gi|219978130	81	11.85	27.48	29.7	23.66	3.85	9.57	8.03	7.25					
AIR12	gi|195654711	27	13.08	6.08	7.69	2.99	4.68	0.32	1.26	0.46	Down	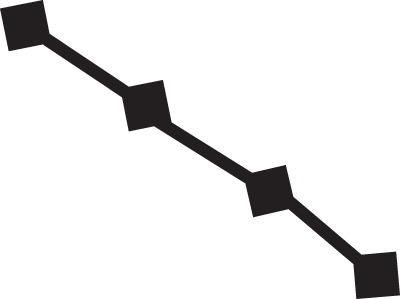			
ATPase subunit 1	gi|102567957	55	197.31	136.131	105.38	92.93	9.14	19.84	4.39	7.07					
Cytochrome P450 CYP72A123	gi|195615656	60	9.87	6.08	2.69	2.26	1.93	0.84	0.38	0.79					
Dolichyl-diphosphooligosaccharide	gi|195648146	70	9.29	6.72	7.95	6.64	0.79	0.58	2.17	0.49					
IQ calmodulin-binding motif family protein	gi|195627120	46	83.63	47.71	38.45	30.95	2.59	1.37	11.15	7.71					
Mitochondrial chaperonin 60	gi|162460375	61	32.59	14.97	12.83	11.44	7.90	3.61	4.26	3.74					
Hypothetical protein	gi|195649497	72	64.36	37.7	35.42	24.72	5.17	6.25	6.38	6.20					
Unknown	gi|219888281	54	39.38	28.96	26.51	25.26	2.97	4.96	3.92	4.64					
Unknown	gi|194702738	47	62.6	52.5	35.7	35.01	6.97	10.70	8.97	8.15					
Unknown	gi|238006974	48	18.55	17.24	14.58	12.36	1.47	4.28	3.21	3.19					
Unknown	gi|194706574	44	13.19	4.94	5.69	4.74	1.43	1.56	0.44	1.35					
Unknown	gi|223947901	76	18.77	13.08	15.16	10.02	5.33	4.56	3.82	2.18					
Unnamed protein product	gi|219902525	61	33	16.34	13.34	11.34	7.64	3.76	4.26	3.34					
Unnamed protein product	gi|158058388	43	16.5	12.36	10.06	6.74	2.12	1.65	1.17	2.21					
Unnamed protein product	gi|219749304	115	18.23	12.07	12.34	10.41	3.15	4.33	1.75	2.92					
Actin-1	gi|195639446	42	33.48	45.11	51.95	56.78	1.33	2.81	6.63	7.04	Up	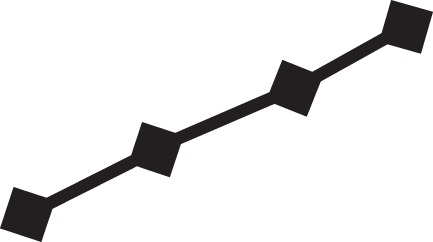			
Lipoxygenase	gi|162461114	98	18	18.67	15.67	26.67	4.94	5.37	1.77	8.46					
Lipoxygenase	gi|162462799	98	11.34	10.67	9.34	17	1.77	3.18	0.33	5.77					
Ribosomal proteinS6	gi|162463315	29	18.14	25.99	28.79	43.48	2.05	3.03	7.68	9.17					
Unknown	gi|219888401	105	22.7	92.651	25.55	161.71	3.67	10.59	14.90	44.88					
Unknown	gi|224029983	78	20.68	39.31	44.17	52.94	4.71	1.85	3.42	18.97					
Unnamed protein product	gi|219748040	105	30.97	72.73	87.33	114.21	5.05	4.09	6.62	34.54					
Unnamed protein product	gi|147223487	32	21	29.02	30.91	41.42	4.13	7.34	5.73	8.57					
Vacuolar ATP synthase catalytic subunit A	gi|195658441	68	32.5	38.38	51.98	55.91	3.69	6.39	9.95	13.16					

*Categories of patterns were assigned based on the means of normalized spectral counts (NSC). Means and standard errors were calculated using the NSC from three biological replicates for each region*.

**Table 2:** **Proteins only present in specific regions in the primary root growth zone**.

Pattern	Identified proteins	Accession number	Molecular weight	R1	R2	R3	R4
Single region present	50S ribosomal protein L1	gi|195625038	44 kDa	+			
	Nucleolar protein 10	gi|195655253	80 kDa	+			
	Phosphoribosylanthranilate transferase	gi|195652617	89 kDa	+			
	Phytochrome A2	gi|129560387	125 kDa	+			
	ATPase family AAA domain-containing protein 1	gi|195637516	41 kDa	+			
	GDP-mannose 4,6 dehydratase 2	gi|195624368	42 kDa	+			
	Guanine nucleotide-binding protein-like 3	gi|195656991	67 kDa	+			
	Hypothetical protein	gi|195647050	69 kDa	+			
	Pescadillo	gi|223945377	71 kDa	+			
	Unknown	gi|194688842	51 kDa	+			
	Unknown	gi|224031377	73 kDa	+			
	Unknown	gi|224030191	48 kDa	+			
	Unknown	gi|238014338	33 kDa	+			
	Unknown	gi|238014320	29 kDa	+			
	Unknown	gi|194689536	80 kDa	+			
	Unknown	gi|219885489	74 kDa	+			
	Unknown	gi|223949449	61 kDa	+			
	Unknown	gi|194690694	59 kDa	+			
	Unknown	gi|194694732	48 kDa	+			
	Unknown	gi|194689188	40 kDa	+			
	Plasma membrane intrinsic protein	gi|162460423	30 kDa				+
	Unknown	gi|226505982	66 kDa				+
	Unknown	gi|194703914	42 kDa				+
	Unnamed protein product	gi|219766771	71 kDa				+
Single region absent	Tubulin beta-1 chai n	gi|135449	50 kDa				−
	Squalene synthase 1	gi|162463458	46 kDa				−
	Hypothetical protein	gi|195612288	60 kDa				−
	Unknown	gi|238011696	60 kDa				−
	Unknown	gi|194707602	41 kDa				−
	5-Methyltetrahydropteroyltriglutamate	gi|195657041	84 kDa	−			
	Beta3-glucuronyl transferase	gi|195612932	37 kDa	−			
	Glycoside transferase, six-hairpin, subgroup	gi|195616232	69 kDa	−			
	Glycosyl transferase	gi|195645114	52 kDa	−			
	HGA4	gi|195625278	58 kDa	−			
	Patellin-5	gi|195624944	58 kDa	−			
	Plasma membrane integral protein ZmPIP2-3	gi|13447805	30 kDa	−			
	Plasma membrane integral protein ZmPIP2-4	gi|13447807	30 kDa	−			
	Plasma membrane MIP protein	gi|162458078	31 kDa	−			
	Proline oxidase	gi|195612286	52 kDa	−			
	Putative auxin efflux carrier	gi|111378666	65 kDa	−			
	Transmembrane protein	gi|162458279	31 kDa	−			
	Transmembrane 9 super family protein member 4	gi|195647402	74 kDa	−			
	Uclacyanin-2 precursor	gi|195610584	20 kDa	−			
	Unknown	gi|194697168	31 kDa	−			
	Unknown	gi|194690676	68 kDa	−			
	Unknown	gi|194690580	25 kDa	−			
	Unknown	gi|223947829	62 kDa	−			
	Unknown	gi|194703616	30 kDa	−			
	Unknown	gi|223949153	35 kDa	−			
	Unknown	gi|219884351	70 kDa	−			
	Unknown	gi|194688782	56 kDa	−			
	Unknown	gi|224033221	40 kDa	−			
	Unknown	gi|223942749	44 kDa	−			
	Unknown	gi|194690662	43 kDa	−			
	Unknown	gi|194690360	58 kDa	−			
	Unknown	gi|224033649	66 kDa	−			
	Unknown	gi|194689110	50 kDa	−			
	Unknown	gi|219885505	53 kDa	−			
	Unnamed protein product	gi|219900697	79 kDa	−			
	unnamed protein product	gi|218405930	64 kDa	−			
Two-region presence	Ankyrin protein kinase-like	gi|195654649	68 kDa	+	+		
	ATP binding protein	gi|195622982	56 kDa	+	+		
	Unknown	gi|223945493	115 kDa	+	+		
	Unknown	gi|219885707	82 kDa	+	+		
	Unknown	gi|194703992	62 kDa	+	+		
	Unknown	gi|194702080	41 kDa	+	+		
	Unknown	gi|223949925	52 kDa	+	+		
	Unnamed protein product	gi|29892201	21 kDa	+	+		
	Unnamed protein product	gi|219731540	91 kDa	+	+		
	Unknown	gi|194689072	49 kDa		+	+	
	Phospho-2-dehydro-3-deoxyheptonate aldolase 1	gi|195642466	59 kDa	+			+
	Unknown	gi|224030621	66 kDa	+			+
	Unknown	gi|194704320	57 kDa			+	+
	Unknown	gi|194706396	45 kDa			+	+

*Region-specific patterns were assigned based on the means of normalized spectral count (NSC). Means and standard errors were calculated using the NSC from three biological replicates for each region*.

## Results and Discussion

### Spatial distribution of relative elongation rate in the primary root growth zone

Kinematic analysis showed that the primary root growth zone encompassed the apical 12 mm (Figure 1A). The relative elongation rate increased as the cells were displaced away from the apex, reaching a maximum between 3–4 mm followed by a gradual decrease to reach zero at about 12 mm. Therefore, the apical 20 mm was divided into four contiguous regions based on the relative elongation rate profile: the 0–3 mm region (R1) that showed acceleration in elongation rate, the 3–7 mm region (R2) that exhibited the initial phase of decelerating elongation rate (>0.2 h^−1^), the 7–12 mm region (R3) of decelerating elongation rate (<0.2 h^−1^), and the 12–20 mm region (R4) that showed no elongation (Figure 1A). These tissues were then used for comparisons of PM-enriched proteomes.

### Plasma membrane enrichment and LC-MS/MS efficacy

A simplified method for PM enrichment based on treatment of microsomal fractions with Brij-58 detergent was recently reported using *Arabidopsis* cell cultures (Zhang and Peck, 2011). The relative efficacy of this PM enrichment strategy was tested in maize root samples by conducting immunoblot experiments with the PM marker AHA (a family of integral PM proton ATPases) and the ER membrane marker SMT-1 (integral ER sterol methyltransferase). Equal amounts of protein from the soluble protein fraction (obtained from the first ultracentrifugation step), crude microsomal fraction, and PM-enriched fraction were separated by SDS-PAGE and blotted with antibodies of AHA1 and SMT-1 (Figure 2A). Comparisons of the CM and PM fractions clearly showed an increase in signal corresponding to AHA proteins in the putative PM-enriched fraction while the abundance of SMT-1 decreased. The presence of the SMT-1 marker in the soluble fraction was unexpected, but we speculated that perhaps the relatively short duration (30 min) of ultracentrifugation was not sufficient to pellet all of the ER fraction. Thus, we investigated if increasing the duration of ultracentrifugation affected the representation of SMT-1 in the microsomal pellet. Indeed, we found that increasing the duration of ultracentrifugation to longer than 30 min increased the proportion of SMT-1 relative to AHA in the microsomal fraction (Figure 2B). Therefore, the 30 min ultracentrifugation was used in all subsequent experiments to decrease ER contamination, thereby increasing the proportion of PM proteins in the sample. These results show that the PM enrichment strategy using Brij-58 detergent contained the highest levels of AHA1 and lowest levels of SMT-1, indicating that the PM fraction was enriched for PM proteins while depleting proteins from the ER. Therefore, the simplified PM enrichment protocol previously described for use in *Arabidopsis* suspension cell cultures (Zhang and Peck, 2011) also is applicable to studies in unrelated tissues such as the maize primary root.

Equal amounts of the PM-enriched protein fractions from the four contiguous regions in the root growth zone were loaded and separated by 1D SDS-PAGE. After separation of samples from a biological replicate, gel slices were excised and digested with trypsin, and the resultant peptide fractions were analyzed by LS-MS/MS analysis for comparisons of PM-enriched proteomes in the different regions of the growth zone (Figure 1B; summary of all raw and processed LC-MS/MS data from each biological replicate is found in Table S1 in Supplementary Material).

### Protein distribution patterns

For analysis of quantitative protein distributions, proteins were considered to be reproducibly identified if they were found in two of the three biological replicates. Comparisons of this subset of reproducible proteins showed that there was a 72% (498/686) overlap between proteins identified from the four contiguous root regions (Figure 2B). R1, which contained the meristem and zone of accelerating elongation rate, had the most unique proteins compared to the other regions (36 in R1 versus 1 in R2, 0 in R3, and 6 in R4). Contiguous regions (R1–3 or R2–4) were more similar than non-contiguous regions (R1 and R4), indicating that the proteomes reflect the developmental gradient of root elongation.

We next analyzed the distribution of proteins in terms of relative abundance throughout the different regions. Of the 686 proteins, 83% (574) did not show any specific pattern of distribution, whereas 6% (43) showed region-distributed patterns (Table 1) and 11% (74) were present in only one or two regions (Table 2). The protein distribution patterns between the four different regions were grouped into several categories (Table 1). For example, when a protein showed lowest expression in R1 and similar levels in R2–4, it was grouped in the category “R1-lowest.” In contrast, if a protein showed highest expression in R1 and decreasing abundance thereafter, it was categorized into the group “down” (see Table 1 for details). Because of the incomplete annotation of the maize genome, a large number of the proteins with defined distribution patterns are listed as “unknown.” However, for some of the annotated proteins, the distribution pattern appears to be consistent with possible biological functions in the growing root, and a few of these examples are discussed below.

### Region-specific changes in aquaporin profiles – role in water movement

**Figure 3:**
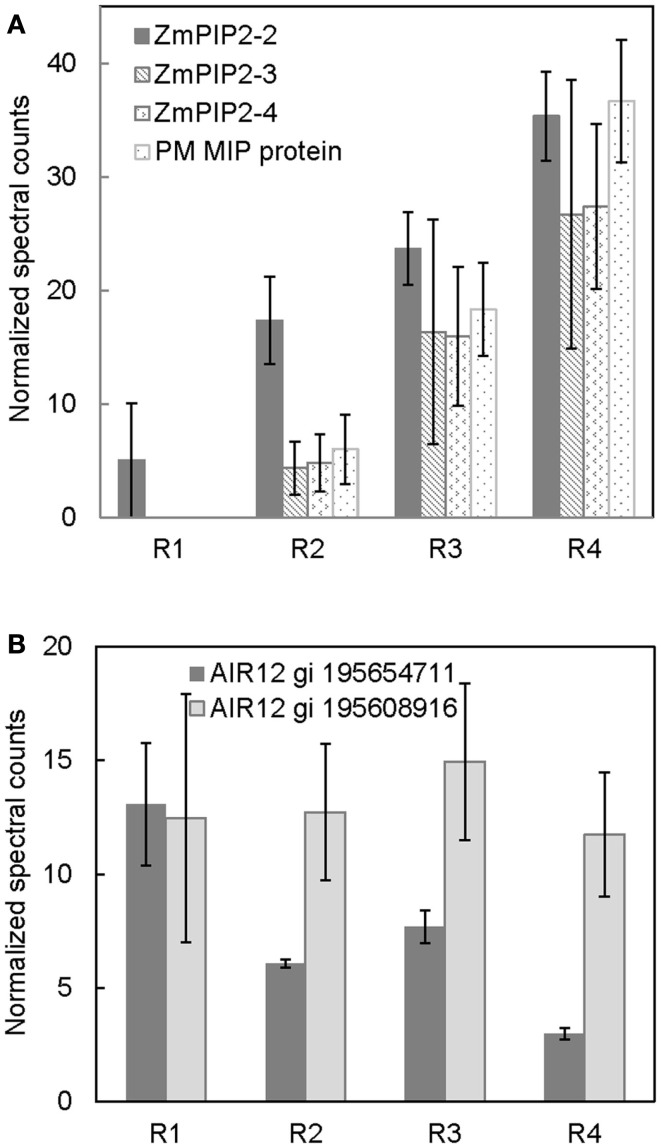
**Spatial distribution of (A) aquaporin protein abundances, and (B) ZmAIR12 protein abundances in R1–4 of the maize primary root harvested 48 h after transplanting to well-watered conditions**. Data are means ± SE of normalized spectral counts from three replicate experiments.

The movement of water in plant tissues occurs by both an apoplastic pathway and a symplastic pathway (Steudle, 2000). The PM intrinsic proteins (PIPs), or aquaporins, are involved in symplastic movement of water across cellular membranes (Maurel and Chrispeels, 2001). Their relative contribution in water movement increases as tissues mature and develop apoplastic barriers such as suberized/lignified cell wall deposits, which reduce apoplastic water movement (Hachez et al., 2006). The results from the present study show a developmental gradient in the abundance of numerous aquaporin proteins (Figure 3A). The protein level of ZmPIP2-2 was lowest in R1 and increased thereafter, reaching a maximum in R4. ZmPIP2-3, 4 and ZmMIP proteins were not detected in R1, whereas there was a progressive increase in their abundance from R2 to R4. The increased aquaporin protein abundance in R4, which represented the maturation region beyond the growth zone (including suberization/lignification of the endodermis and development of the casparian strip), is likely involved in increased symplastic water transport. The abundance pattern of aquaporins observed in this study is similar to the aquaporin abundance patterns observed previously using immunoblot analyses in maize primary roots by Hachez et al. (2006). Therefore, our quantitative PM proteomic comparison is consistent with known spatial gradients of PM proteins, supporting the validity of this strategy.

### Spatial distribution of AIR12 proteins – potential role in redox signaling

Two apparent maize orthologs of *Arabidopsis thaliana* AIR12 (for auxin induced in root cultures) were identified in all four regions of the root (Figure 3B). One of the ZmAIR12 proteins (gi 195608915) was ubiquitously distributed across all four regions, whereas another ZmAIR12 (gi 195654711) was more abundant in R1 compared to R2–4. An ortholog of AtAIR12 in soybean was recently identified as the major PM-localized b-type cytochrome that is fully reduced by ascorbate and fully oxidized by monodehydroascorbate radicals (Preger et al., 2009). The AtAIR12 and GmAIR12 proteins were found to be highly glycosylated and contained a glycosylphosphatidylinositol-anchor that positioned the proteins on the external side of the PM *in vivo* (Borner et al., 2003; Preger et al., 2005, 2009). AIR12 is physically associated with other redox signaling proteins and is suggested to be a link between the apoplast and cytoplasm in redox signaling (Lefebvre et al., 2007; Preger et al., 2009). Therefore, higher levels of ZmAIR12 (gi 195654711) in R1 of the maize primary root may indicate increased redox signaling, which would be consistent with increased apoplastic superoxide production in this region that has been suggested to play a role in cell wall loosening activities (Liszkay et al., 2004). In addition, increased superoxide production in the apical region of primary roots of *Arabidopsis* was suggested to be involved in regulating meristem size (Tsukagoshi et al., 2010). If ZmAIR12 activity is involved in regulating cell expansion, it will be interesting to understand how this process is regulated as one member of the protein family is uniformly distributed in all regions whereas another shows region-distributed accumulation.

### Spatial distribution of cell wall biosynthesis related proteins

#### Cellulose synthases

**Figure 4:**
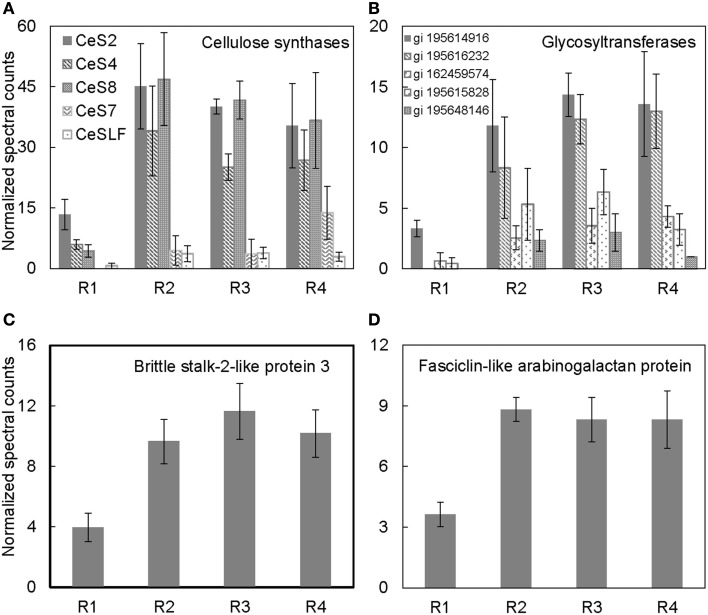
**Spatial distribution patterns of protein abundances for (A) cellulose synthases, (B) glycosyltransferases, (C) brittle stalk-2-like protein 3, and (D) fasciclin-like arabinogalactan proteins in R1–4 of the maize primary root harvested 48 h after transplanting to well-watered conditions**. Data are means ± SE of normalized spectral counts from three replicate experiments.

Cellulose, the main constituent of plant cell walls, is composed of parallel unbranched glucan chains referred to as cellulose microfibrils (Anderson et al., 2010). These microfibrils are synthesized by large multimeric complexes of cellulose synthase proteins at the PM (Somerville, 2006). As cells expand, there is increased deposition of cellulose synthase complexes at the PM, and these complexes in turn deposit cellulose necessary for maintaining rigidity and directional growth of the cells (Baskin, 2005). Several cellulose synthases were identified in the PM-enriched fraction from the different regions of the maize primary root (Table 1). The abundance of cellulose synthases was lowest in R1, increased in R2, and remained high in R3–4 (Figure 4A). Because the integrated expansion of the cells in the maize primary root (as determined by the area under the relative elongation rate curve) is maximal in R2–3, the increased abundance of cellulose synthases in these regions is consistent with increased cell wall deposition in the elongating cells. As R4 corresponds to the region of maturation and secondary cell wall synthesis (Figure 1A), an increased abundance of cellulose synthases in this region is consistent with increased cell wall maturation (Figure 4A).

#### Glycosyltransferases

Several glycosyltransferases were differentially distributed throughout the regions of the root (Figure 4B). Glycosyltransferases are enzymes that transfer a sugar moiety from activated donor to acceptor molecules forming glycosidic bonds (Zhong and Ye, 2003; Lim and Bowles, 2004). Plant glycosyltransferases are involved in biosynthesis of several cell wall components such as polysaccharides, hemicelluloses, and pectins, and these enzymes may also be involved in transferring sugar molecules to proteins, hormones, and secondary metabolites (Zhong and Ye, 2003; Liepman et al., 2010). Therefore, the increased abundance of various glycosyltransferases particularly in the regions of maximal cell expansion (R2–3; Figure 1A) and secondary cell wall formation (R4) indicates that the glycosyltransferases identified in this study may be involved in cell wall synthesis (Figure 4B). Although some studies have suggested that glycosyltransferases are associated with endomembranes (Keegstra, 2010), the localization of cellulose synthases, a major family of glycosyltransferases, at the PM suggests that glycosyltransferases can be associated with the PM (Scheible and Pauly, 2004; Somerville, 2006). Additionally, a recent study of PM proteomics in poplar in which the PM fraction was obtained from two-phase partitioning of microsomal membranes identified several glycosyltransferases (Nilsson et al., 2010). Therefore, it is likely that different classes of glycosyltransferases are localized to different compartments dependent on their function(s).

#### Brittlestalk-2-like protein 3 and fasciclin-like arabinogalactan proteins

The orientation of microfibrils is known to regulate cell shape. In isotropic expansion, the microfibrils are oriented in all directions, whereas in anisotropic expansion they are oriented perpendicular to the direction of expansion (Baskin, 2005). Although expansion is anisotropic in the root growth zone, the degree of anisotropy increases as the cells move away from the root apex (Liang et al., 1997; Baskin et al., 2004). These changes potentially involve changes in cellulose microfibril orientation and patterning. Several studies have shown that the cellulose microfibril orientation can be modified by PM-localized proteins such as COBRA and fasciclin-like arabinogalactan proteins in *Arabidopsis* and an ortholog of COBRA in maize, brittlestalk-2 (Roudier et al., 2005; Ching et al., 2006; MacMillan et al., 2010).

*Brittlestalk-2* (*bk2*) was identified in a screen for maize mutants defective in cellulose biosynthesis; the mutant stalks had reduced mechanical strength (Ching et al., 2006). Analysis of the brittle phenotype found that disruption in the *bk2* gene interferes with the pattern of cellulose microfibril deposition, which leads to reduced cellulose and increased lignin in the *bk2* mutant shoot tissues (Ching et al., 2006; Sindhu et al., 2007). In *Arabidopsis*, the ortholog of *bk2*, *COBRA*, encodes a GPI-anchored PM-localized protein that is suggested to regulate cellulose microfibril deposition and anisotropic expansion in both primary root and hypocotyl tissues (Roudier et al., 2005). The protein abundance pattern of brittlestalk-2-like protein (Figure 4C) is similar to the mRNA expression pattern of *COBRA* in the primary root (Roudier et al., 2005), suggesting that brittlestalk-2-like protein may be involved in cellulose microfibril patterning and anisotropic expansion in the maize primary root.

Similarly, fasciclin-like arabinogalactan proteins are suggested to be involved in cellulose microfibril orientation and secondary cell wall synthesis in *Arabidopsis* and *Eucalyptus* (MacMillan et al., 2010). These proteins belong to a large protein family containing the cell adhesion fasciclin domain, which is also conserved in cell adhesion proteins in bacteria, algae, fungi, and animals (Johnson et al., 2003). In plants, the fasciclin-like arabinogalactan proteins are suggested to be PM-localized either with or without a GPI-anchor (Borner et al., 2003; Johnson et al., 2003; Lefebvre et al., 2007). However, the functions of the various fasciclin-like arabinogalactan proteins in plants are still largely unknown. Recent analysis revealed that mutations in two stem-specific fasciclin-like-arabinogalactan proteins lead to altered cellulose deposition and reduced stem stiffness in *Arabidopsis* stems (MacMillan et al., 2010). Thus, the increased accumulation of fasciclin-like-arabinogalactan protein in R2–4 (Figure 4D) hints at a role for this protein in modification of cellulose microfibril deposition.

## Conclusion

The results in the present study demonstrate that the simplified PM enrichment method previously shown to work in *Arabidopsis* also can be used to characterize the developmental distribution of PM proteins in the maize primary root. Therefore, it appears that this technical strategy may be more broadly applicable to PM protein studies in diverse plant species. In addition, we found that shorter durations of ultracentrifugation decrease the representation of the ER marker, SMT-1, in the microsomal fraction. We hypothesize that this difference is caused because of the differences in buoyant densities of PM vs. ER-containing vesicles in maize root extracts. However, it should be noted that this method does not replace more stringent, but challenging, methods such as two-phase partitioning if the goal of the study is to more conclusively demonstrate that a protein specifically localizes to the PM. Rather, the present method serves as a simple and robust method to increase the representation of PM proteins in proteomic studies.

Of the proteins showing defined developmental distribution patterns in the primary root of maize, a number of them, such as the aquaporins and cellulose synthases, appear logical in relation to their roles in cell expansion. Therefore, other proteins of unknown function such as the glycosyltransferases and the fasciclin-like arabinogalactans serve as candidates for involvement in growth regulation. Of course, further characterization studies are needed to determine the functional significance of their differential distribution in the growth zone of the maize primary root.

## Conflict of Interest Statement

The authors declare that the research was conducted in the absence of any commercial or financial relationships that could be construed as a potential conflict of interest.

## Footnotes

^1^http://www.thegpm.org

^2^http://www.ncbi.nlm.nih.gov/protein

## Supplementary Material

Supplementary Table S1 **Complete list of all raw and normalized spectral counts for proteins identified in this study**.
